# Brief review: Cardiac complications and platelet activation in COVID-19 infection

**DOI:** 10.7196/AJTCCM.2020.v26i3.107

**Published:** 2020-09-16

**Authors:** C Feldman, R Anderson

**Affiliations:** 1 Department of Internal Medicine, Faculty of Health Sciences, University of the Witwatersrand, Johannesburg, South Africa; 2 Department of Immunology, Faculty of Health Sciences, University of Pretoria, South Africa

**Keywords:** COVID-19, Cardiovascular events, Pathogenesis, Platelets

## Abstract

COVID-19 pneumonia, much like that of bacterial and viral community-acquired pneumonia before it, is accompanied by a high rate of cardio- and cerebrovascular events that are associated with an increased risk of complications and a greater mortality. Although the mechanisms underlying the pathogenesis of these adverse events are not entirely clear and may be multifactorial, platelets appear to have a prominent aetiologic role and this, together with an overview of the clinical evidence, forms the basis of this short review.

## Background

A number of studies have documented significant declines in the incidence of acute myocardial infarction, particularly the more serious, ST-segment elevation-type myocardial infarction, as well as its mortality rate, over the past two decades.^[1–4]^ While the reasons may be multifactorial, collectively these studies suggest that primary prevention efforts, the use of various drugs, including beta-blockers, angiotensin-converting enzyme inhibitors and also anti-platelet therapies, as well as broader use of reperfusion and additional adjunctive therapies are important. However, more recent research has focused on the occurrence of myocardial infarction as a complication of infections, particularly community-acquired pneumonia (CAP), and this has become especially highlighted by findings originating from the current severe acute respiratory syndrome coronavirus-2 (SARS-CoV-2) pandemic.

Recent studies,^[5–10]^ including systematic reviews and meta-analyses,^[11,12]^ and various reviews of the literature,^[13–19]^ have highlighted the presence of underlying cardio- and cerebrovascular comorbidities, as well as the occurrence of acute cardio- and cerebrovascular events (CVEs) in patients with SARS-CoV-2 infection (COVID-19 disease). These are similar to those previously described in patients with severe acute respiratory syndrome (SARS) and Middle East Respiratory Syndrome (MERS), and, even earlier, in patients with other viral infections, especially influenza.^[19]^ While both the initial and subsequent studies of COVID-19 infection documented various cardiovascular (CV) conditions to be risk factors for COVID-19 pneumonia, some of the subsequent studies recognised that these comorbidities, as well as the acute cardiac events that occurred during these infections, were also risk factors for more severe disease/critical illness and were predictors of mortality.^[5-7,11,12]^ For example, one study of COVID-19 patients indicated that the proportions of hypertension and cardio-cerebrovascular disease were 17.1 and 16.4%, respectively, and the incidences were approximately two- and three-fold higher in ICU/severe cases compared with non-ICU/non-severe cases.^[12]^

## Cardiac complications

With regard to acute cardiac complications, this latter study indicated that acute cardiac injury occurred in at least 8% of patients with COVID-19, the incidence of which was 13-fold greater in ICU/severe cases compared with non-ICU/non-severe cases, and clearly impacted on prognosis.^[12]^ A second study indicated that 19.7% of COVID-19 cases were documented to have had cardiac injury, and compared with cases without cardiac injury, the former cases were older, had more comorbidities, had laboratory features suggestive of more severe infection (higher white cell count, C-reactive protein (CRP), procalcitonin (PCT)), as well as higher levels of cardiac biomarkers, including creatinine kinase (CK)-MB, highly sensitive troponin I, and N-terminal pro-hormone brain natriuretic peptide (NT-pro-BNP) levels, among other factors.^[7]^ The need for non-invasive or invasive mechanical ventilation was higher in those with cardiac injury compared with those without, as were complications such as acute respiratory distress syndrome (ARDS), acute kidney injury (AKI), and coagulation disturbances, while these patients also had a higher mortality.^[7]^ A third study noted that myocardial injury, characterised as cardiac dysfunction and arrhythmias, occurred in 27.8% of a cohort of 187 patients with COVID-19, and was significantly associated with a fatal outcome compared with those patients without myocardial injury.^[6]^

Overall, the acute cardiac events that have been documented to occur in patients with COVID-19 infection included acute coronary syndrome, arrhythmias and acute heart failure,^[15,19,20]^ much like that of all-cause CAP and pneumococcal CAP, as well as influenza infections, especially pandemic infections, as has been described elsewhere.^[14,20,21]^ Additional events that have been described include myocarditis, including typical acute myocarditis, as well as myocarditis presenting as reverse Tako-Tsubo syndrome,^[8]^ and also cardiogenic shock and death.^[15]^ Nevertheless, there has been some debate in the literature as to the true incidence of these cardiac events in patients with COVID-19 infections, as there have been few reports of echocardiographic criteria of cardiac injury. To date, reliance on the diagnosis of cardiac injury has been based mainly on serum cardiac biomarkers that cannot differentiate between different types of cardiac injury, and which may be raised in conditions such as septic shock and in secondary bacterial infection without the presence of cardiac injury; furthermore, it has also been proposed that demonstration of the true attributable mortality due to cardiac injury in COVID-19 infection is lacking.^[22,23]^

Clearly, we need more investigation of the types, mechanisms and treatment of these cardiac events, in order to understand and manage them better.^[22,23]^ Additional thromboembolic events that have been described, over and above the cardiac events of acute coronary syndrome/acute myocardial infarction, include both arterial and venous events, most commonly acute pulmonary embolism,^[9,10,13]^ as well as deep venous thrombosis, upper limb venous thrombosis, peripheral arterial thrombosis, stroke and various others.^[13]^ While it appears clear that these cardio- and cerebrovascular events are associated with acute implications, there is also concern, based on data from survivors of other causes of CAP, and in follow-up studies of the other coronaviruses, including SARS-CoV and MERS-CoV, that there may also be long-term implications associated with persistent heightened inflammatory responses and procoagulant activity after resolution of the CAP symptoms.^[16,18]^

Initially it was suggested that the mechanisms of the cardiac events may be related to interactions of the SARS-CoV-2 virus with the angiotensin-converting enzyme-2 (ACE2) receptor, since both this virus and the SARS virus, and influenza before that, were documented to use the ACE2 receptor as a functional receptor; furthermore, smoking and hypertension (for which ACE inhibitors and ACE2 receptor blockers are commonly used as treatment) have previously been documented as underlying comorbid risk factors for COVID-19 infection and both have been shown to increase expression of the ACE2 receptor.^[14,24,25]^ However, the data for this were reviewed and several societies issued strong recommendations against the cessation of these antihypertensive medications until more information was available, a contention supported by a special report published in the *New England Journal of Medicine*. ^[26]^ A subsequent study from Wuhan also discounted the use of these agents as either a risk factor for severe disease or mortality in patients with COVID-19 infection.^[25]^

Another potential cause of cardiac complications, particularly arrhythmias, such as ventricular tachycardia, was considered to be the use of certain drugs, either alone or in combination, for the prophylaxis or treatment of COVID-19 infection, especially chloroquine/hydroxychloroquine and azithromycin due to their effects on the QT interval.^[27]^ A number of additional possible mechanisms for these CVEs have been suggested in various studies and reviews, including excessive systemic inflammatory responses, myocardial depression by pro-inflammatory cytokines, immobilisation, hypoxia with decreased oxygen delivery to the heart at the time of increased oxygen demand resulting in ischaemia, direct vascular infection with inflammation, and direct myocardial infection, among many other possibilities.^[18,19]^ There is also emerging evidence that abnormalities of the haematological system, including effects on platelets and hypercoagulability, may play a role in the pathogenesis of CVEs in patients with COVID-19 infection and that anticoagulant treatment could be associated with decreased mortality in COVID-19 infection.^[19,28,29]^

## Platelets and COVID-19

An accumulating body of evidence has implicated platelets in the pathogenesis of SARS-CoV-2 infection. However, the role played by these cells, be it protective, harmful, or even both, remains unresolved. Given this uncertainty, the remaining sections of this review are focused on: *(i)* the role of platelets in antiviral host defence, specifically in the context of infection caused by single-stranded RNA viruses; *(ii)* the effect of overwhelming viraemia on platelet numbers and function in COVID-19; and *(iii)* the circulating platelet count as a possible determinant of the resilience of young children, as well as the vulnerability of the elderly and those with comorbidities, to development of severe, life-threatening COVID-19.

### Platelets in host defence against single-stranded RNA viral pathogens

Platelets have been identified as key players in antiviral host defence by trapping, internalising and exposing viral pathogens to various antiinfective peptides/polypeptides, as well as via recruitment and activation of cells of the innate and adaptive immune systems.^[30–33]^ Recognition of viral pathogens is mediated predominantly via expression of pattern recognition receptors on platelets that interact with viral nucleic acid and glycoproteins. In the case of SARS-CoV-2 and other singlestranded RNA viruses, toll-like receptor 7 (TLR7) plays a key role in pathogen capture.^[34–36]^ Systemic exposure of platelets to viral single-stranded RNA was revealed in an earlier study on MERS coronavirus, encompassing 21 hospitalised patients.^[37]^ The authors detected viral nucleic acid in the blood of 33% of patients at the time of initial diagnosis that was predictive of disease severity,^[37]^ a finding indicative of the involvement of platelets in the early containment of viremia.

In this latter context, evidence in support of a beneficial role for platelets in protecting against COVID-19 is derived from a number of studies that have described an association between thrombocytopenia and severe fatal disease.^[29,38-43]^ Putative mechanisms that may underpin COVID-19-associated thrombocytopenia include the following:

  **Inhibition of megakaryocyte proliferation.** In this setting, elevated levels of type I antiviral interferons (IFNs) have been reported to inhibit the proliferation of megakaryocytes.^[44,45]^ However, given that severe SARS-CoV-2 infection has been associated with significant impairment of the production of type I IFNs,^[46–48]^ it seems unlikely that this mechanism is operative.

**Cytocidal effects of SARS-CoV-2 on platelets.** Although plausible, no evidence exists (to our knowledge) that supports this mechanism of SARS-CoV-2-associated thrombocytopenia. In this context it is noteworthy, however, that human immunodeficiency virus 1 (HIV-1), which is also a single-stranded RNA virus, is not only internalised by platelets, but can also replicate in these cells.^[49]^ This mechanism is operative in virally-suppressed, HIV-1-infected subjects and may represent a means of viral persistence and transmission.^[49]^

**Hyperactivation of platelets by SARS-CoV-2.** Infection-related thrombocytopenia may also result from hyperactivity of platelets that leads to the formation of large, intravascular aggregates of these cells, both homotypic and heterotypic, resulting in significant decreases in circulating platelet counts. Such a scenario has been described following infection of both humans and mice with another single-stranded RNA virus, viz. encephalomyocarditis virus (cardiovirus A), that resulted in the formation of large, intravascular platelet:neutrophil aggregates and thrombocytopenia.^[34]^ In this setting, hyperactivation of platelets is mediated via interaction of TLR7 expressed on these cells with viral single-stranded RNA.^[34]^

Evidence in support of the existence of this type of mechanism in COVID-19 is derived from a very recent study describing the presence of high levels of biomarkers of neutrophil extracellular traps (NETs) in the blood of patients hospitalised with severe COVID-19 receiving mechanical ventilation.^[50]^ One of these biomarkers of NETosis, citrullinated histone H3, correlated positively with platelet counts (r=0.45; *p*<0.0001).^[50]^ These findings are in keeping with the involvement of activated platelets in driving formation of NETs,^[51]^ which have been implicated in the pathogenesis of organ damage and mortality in COVID-19.^[52]^

Another mechanism by which platelet dysfunction may contribute to organ damage relates to the key role of platelets in maintaining the structural integrity of the endothelial barrier. In this context, platelets prevent vascular leakage at sites of neutrophil extravasation by binding to endothelial von Willebrand factor by a mechanism that involves strengthening of cortical actin bundles.^[53]^ This mechanism is distinct from that involved in platelet-mediated protection against inflammatory bleeding at sites of neutrophil extravasation.^[53]^

The aforementioned mechanisms and consequences of systemic hyperactivation of platelets are likely to contribute to the thrombotic and microangiopathic pathology described in the lungs of African American patients who succumbed to COVID-19.^[54]^

Notwithstanding mechanisms secondary to severe pulmonary dysfunction, platelets may also contribute to the development of the CV complications of COVID-19 via: *(i)* translocation of the virus from the blood to the heart either by active transport of bound/internalised viral particles and/or via microvascular leakage, predisposing to development of viral myocarditis; and *(ii)* coronary microvascular obstruction due to intravascular platelet aggregation and NETosis.

### Platelets and age-related severity of COVID-19 in children

**Fig. 1:**
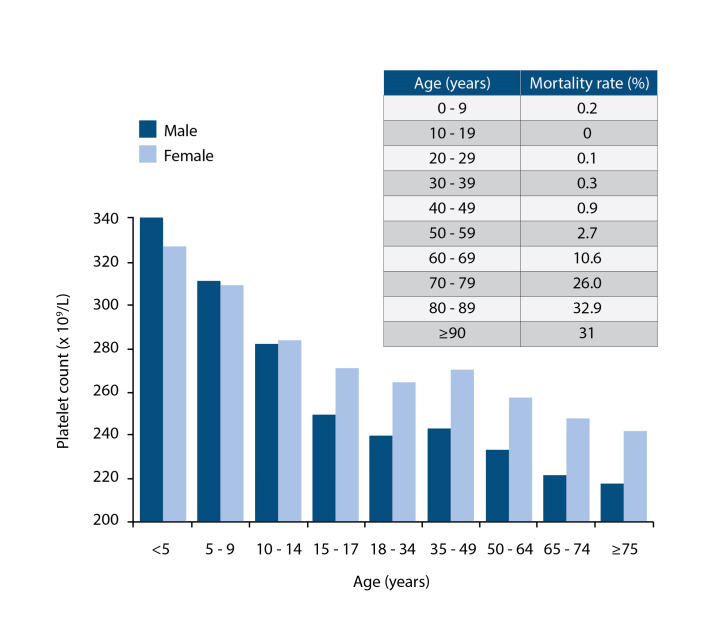
Platelet count by age in the examined populations, including categorisation according to gender. Reproduced with the approval of the authors, Biino *et al*. ^[56]^ Superimposed on the platelet counts are the age-related mortality data for Italy as of 15 June 2020.^[57]^

Notwithstanding those who develop multisystem inflammatory syndrome, children appear to develop a milder form of COVID-19 than their older counterparts.^[55]^ While the reasons that underpin the lower COVID-19 mortality rates in children are likely to be multifaceted, one potential contributor is the notable distinction between children and older adults with respect to the platelet count and age. In this context, a study reported by Biino *et al*.^[56]^ measured circulating platelet counts in relation to age (range <5 - ≥75 years) in male and female inhabitants (*N*=40 987) of seven areas in Italy, is particularly noteworthy. Those in the younger age groups had the highest circulating platelet counts (299 × 10^9^ v. 252 × 10^9^ /L) when comparing counts for those aged <15 years with those in the 15 - 64 years of age group, respectively (*p*<0.001) (Fig. 1).^[56]^ An apparent association between age, platelet counts and COVID-19-related mortality rates is evident by superimposition of Italian COVID-19 mortality data in relation to age as of 15 June 2020 (237 000 cases; overall mortality of 14%) ^[57]^ on the platelet count data (Fig. 1). Although intriguing, we do concede that this association may be coincidental.

In addition, the decline in the numbers of circulating platelets associated with advancing age is also accompanied by acquisition of a pro-inflammatory/pro-thrombotic phenotype by these cells that may predispose the elderly,^[58,60]^ particularly African Americans,^[61]^ to severe COVID-19 infection. Type 2 diabetes, which carries a high risk for severe COVID-19 infection, is also associated with a hyperactive platelet phenotype.^[62]^

Clearly, acute CVEs represent a major cause of morbidity and mortality in patients with severe COVID-19 infection. Although the exact mechanisms underpinning the pathogenesis of these adverse events are unresolved, platelets appear to play a prominent role, which if verified, may identify these cells as potential therapeutic targets. Although beyond the brief of this short review, it is important to note that severe COVID-19 infection is becoming increasingly recognised as a multisystem vasculopathy that may require treatment, in its own right.^[63]^ To this end a number of guidelines have been published addressing anti-thrombotic therapy in the management of COVID-19 infection.^[64]^ Notwithstanding the necessity for stringently controlled clinical trials and awareness of the risk of bleeding complications, dual antiplatelet-targeted therapy with aspirin (thromboxane A_2_ inhibitor) and clopidogrel (ADP-targeted P_2_Y_12_ receptor antagonist) represents a potentially effective option in COVID-19.^[65]^ In this context an ongoing clinical trial, NCT04333407, is investigating the effects of aspirin, clopidogrel and rivaroxaban (oral thrombolytic factor Xa inhibitor), together with atorvastatin and omeprazole, on all-cause 30-day mortality after hospital admission, as well as serial measurement of serum cardiac troponin in patients with COVD-19 as a strategy to prevent these cardiac complications.^[66]^ Innovative studies of this type are clearly necessary to identify effective therapies.
